# Calibration of piezo actuators and systems by dynamic interferometry

**DOI:** 10.3762/bjnano.16.143

**Published:** 2025-11-17

**Authors:** Knarik Khachatryan, Michael Reichling

**Affiliations:** 1 Institut für Physik, Universität Osnabrück, Barbarastr. 7, 49076 Osnabrück, Germany

**Keywords:** cantilever excitation, fiber interferometer, NC-AFM, piezo calibration, non-contact atomic force microscopy

## Abstract

To achieve precise measurements of small displacements in non-contact atomic force microscopy, it is crucial to control the position of moving parts with high accuracy. This is commonly accomplished by piezo actuators, for instance, in the form of tube piezos for positioning the tip or optics. For their calibration, we propose an approach based on the dynamic response signal from a fiber interferometer used for cantilever displacement detection. The fine-positioning *z*-piezo of the fiber is calibrated by the analysis of measurements of the dynamic interferometer response signal recorded for various cantilever oscillation amplitudes and varied distances between the cantilever and the fiber end. Furthermore, we demonstrate the cantilever oscillation amplitude calibration under conditions of various amounts of tube piezo contraction and extension. The merits and limits of accuracy for such type of calibration are discussed.

## Introduction

Interferometric displacement detection stands as a cornerstone in high-precision techniques employed in cantilever-based atomic force microscopy (AFM), since its early days [1–6]. This method of cantilever displacement detection is specifically well suited for non-contact atomic force microscopy (NC-AFM) operation in an ultrahigh-vacuum (UHV) environment at low temperature and has been developed tremendously over the last three decades [7–12].

In frequency-modulated NC-AFM, the cantilever is kept at oscillation with constant amplitude, yielding an interferometric signal that is a periodic function of time. However, it is generally not a harmonic oscillation due to the convolution of the (quasi)-harmonic oscillation of the cantilever with the spatially modulated light field in the interferometer cavity [13]. Dynamic interferometric signals have been studied in the context of NC-AFM using the interferometric concepts of Michelson and Fabry–Perot interferometers [14–15]. Both interferometers rely on the precise alignment of a single-mode optical fiber delivering the light and receiving the optical signal generated in the optical cavity formed by the cantilever and the fiber end. Controlling and stabilizing the fiber–cantilever distance is of principal importance for the accurate operation of the interferometric detection system [16]. Here, we address the aspect of calibrating the *z*-motion of the fiber tube piezo [17–18] with high accuracy to ensure full control over the interferometer cavity. Measurements involve the expansion and contraction of the piezo tube by an amount of the order of 100 nm, raising the issues of piezo nonlinearity [19], hysteresis [20], and creep [21]. Therefore, we address systematic errors in tube piezo calibration and explore to what extent the cantilever amplitude calibration [13] is affected by the actual extension or extension history of the tube piezo.

## Experimental

Experiments are performed with a NC-AFM interferometric setup, and the methods of interferometer signal analysis are as described in [13]. The schematic setup of the system electronics and relevant voltages is detailed in Figure 3 of [16]. We investigate the dynamics of the free cantilever excited to oscillation at constant amplitude *A* and frequency *f*_exc_, which is always kept at the fundamental cantilever eigenfrequency *f*_0_.

Initially, the interferometer is stabilized so that the working point is the inflection point and center of symmetry of the time-dependent interferometer signal [16], implying that the mean cavity length is *d*_0_ = 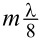, where *m* is an odd integer and λ is the vacuum wavelength of the light used for interferometer operation in a UHV environment. As long as the respective stabilization loop of interferometer alignment is active [16], this adjustment is maintained even in the presence of drift or piezo creep by the automatic adjustment of the voltage applied between the fiber tube piezo inner electrode and the common potential of the tube piezo voltage sources 
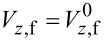
. To contract or expand the tube piezo, the automatic control loop action is frozen and an appropriate voltage 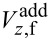 is added to 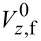, yielding an incremental change *d*_err_ of the mean cavity length to 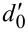 = *d*_0_ + *d*_err_. This quantity can be extracted from the time-domain interferometric signal by a two-step fit method based on Equation 1 as detailed in [13].


[1] $$V_{\text{sig}}=V_{\text{DC}}+V_{0}\sin\left(\frac{4\pi }{\lambda }\left({d^{\prime }}_{0}\;-\;A\sin\left(2\pi \;f_{\text{exc}}\;t\;-\;\varphi \right)\right)-\frac{\pi }{2}\right).$$

Here, *V*_DC_ represents the DC part of the interferometer signal voltage, *V*_0_ is the voltage amplitude of the modulated signal, and φ is the phase shift introduced by the electronics in the signal path, which may be determined from the fit. Experiments are performed with laser light of wavelength λ = λ_vac_ = 780.41 nm and the excitation frequency is kept at the cantilever eigenfrequency *f*_exc_ = 169.667 kHz determined at the beginning of the measurements.

The piezoelectric tube (PZT-8, EBL Products Inc., East Hartford, CT, USA) used for fiber positioning is specified with a piezoelectric constant *d*_31_ = −0.95 Å/V [22] at 293 K, according to the manufacturer’s data sheet [23]. The extension or contraction of the fiber tube piezo Δ*L* depends on *V_z,_*_f_ and the dimensions of the tube [24].


[2] $$\Delta L=d_{31}\frac{L}{h}V_{z,\text{f}},$$

where *L* and *h* are the length of the piezo tube and the wall thickness, respectively. According to the widely used convention, the piezo tube is poled so that a negative potential applied to the inner electrode *V_z,_*_f_ *<* 0 results in an extension Δ*L >* 0 that translates into a reduction of the cavity length Δ*d* = *d*_err_ = −Δ*L <* 0. From *d*_31_ and the geometry parameters of the tube piezo (*L* = 31.8 mm and *h* = 1.40 mm), we deduce a nominal fiber tube piezo calibration factor of 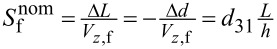 = −2.15 nm/V relating the extension or contraction Δ*L* of the tube piezo to the applied voltage.

### Fiber tube piezo calibration

In our measurements, the fiber is mounted at α = 15° so that it is directed perpendicular to the cantilever, which is also mounted at the same angle α to the horizontal [25]. The vertical movement of the fiber in *z*-direction (*z_f_*) changes the cavity length with 
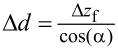
. For the measurement of the actual tube piezo calibration factor 
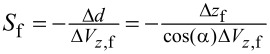
, we measure the contraction and extension of the tube via the change in cavity length Δ*d* as a function of the change in the added voltage 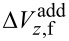. Initially, the control loop is frozen at 
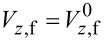
, with *d*_err_ = 0 for a long time (many days) to facilitate complete equilibration of the piezo tube. After the start of the measurement, 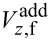 is varied in positive and negative directions in steps of 20 V, while an accommodation time of 10 min passes before the extension or contraction is measured. The cantilever excitation voltage is kept constant at *V*_exc_ = 3.50 V, yielding an oscillation amplitude of *A* = 111 nm, facilitating convenient interferometer signal fits.

## Results and Discussion

**Figure 1:**
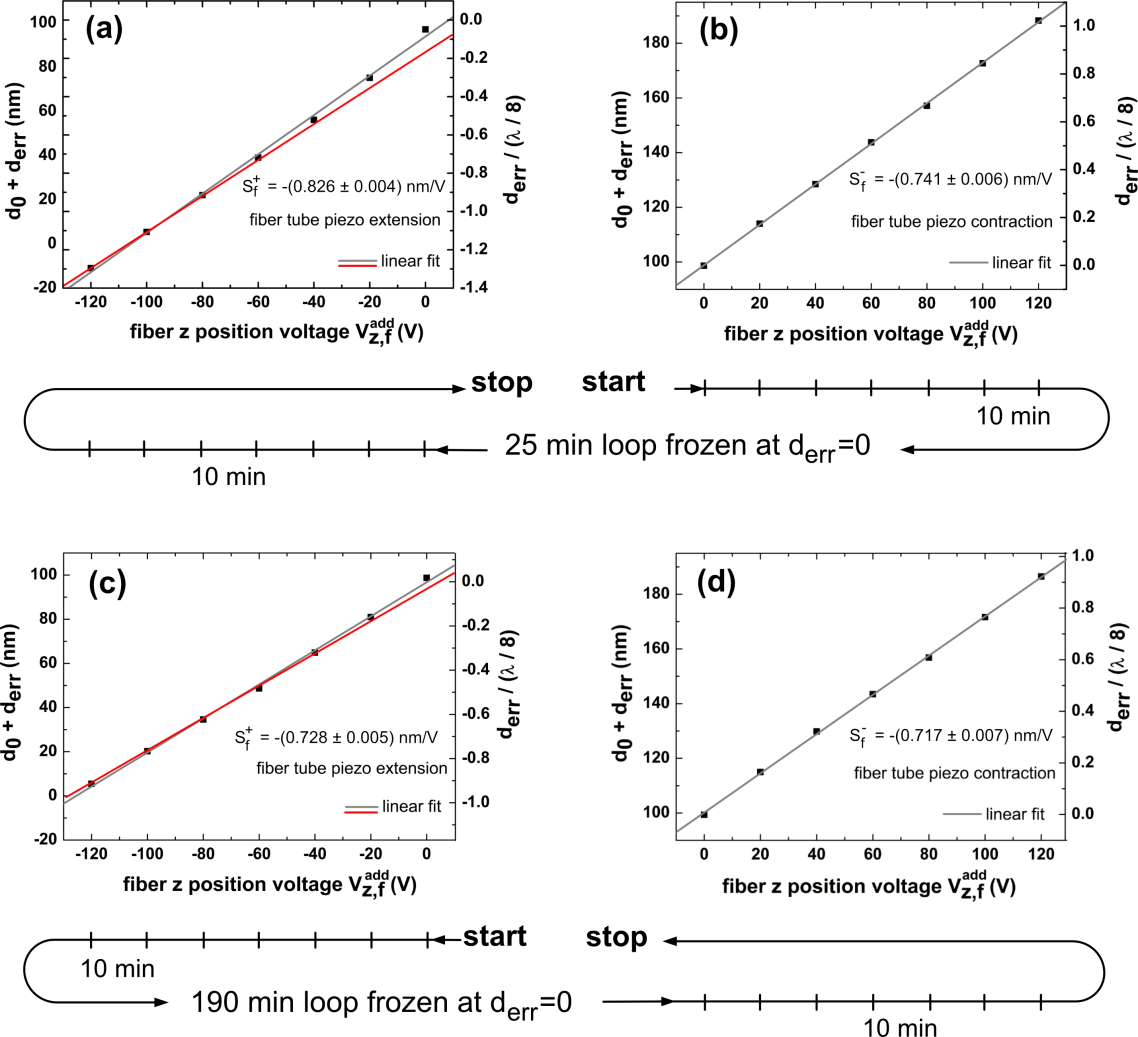
Fiber tube piezo *z*-movement calibration measured with a cantilever oscillation amplitude of (a, b) *A* = 111 nm and (c, d) 126 nm. (a, c) Displacement measurements for tube extension (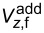 *<* 0, Δ*L >* 0). (b, d) Displacement measurements for tube contraction (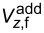 *>* 0, Δ*L <* 0).

Results are compiled in the four frames of Figure 1, including linear functions that fit the measurement points. Measurements have been performed in the sequence of contraction (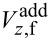 = 0 … 120 V, Figure 1b) and then extension (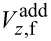 = 0 … −120 V, Figure 1a). Note that the contraction measurement yields a perfect result with the straight line very well fitting the data and crossing (0,0) in the (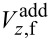, *d*_err_) plane, while the fit for the following expansion measurement (Figure 1a) is less precise and exhibits a systematic trend of approach towards a limiting straight line defined by the last three data points (red line in Figure 1a). We find that the calibration factors for contraction (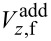 *>* 0) and extension (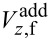 *<* 0) reproducibly differ from each other. From the slope of the straight lines in Figure 1, we determine 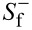 = −(0.741 ± 0.006) nm/V for the contraction and 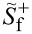 = −(0.889 ± 0.020) nm/V for the extension. Evaluating the calibration factor from the red, straight line yields 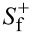 = −(0.826 ± 0.004) nm/V, a value closer but clearly different from 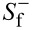. Further, we note that, in Figure 1a, neither the first data point of this measurement nor one of the fit lines crosses (0,0). We attribute this behavior to piezo creep and hysteresis occurring after switching polarity from positive to negative. Note that we observe this phenomenon although we paused the measurement for 25 min between contraction and extension experiments to give the system time for equilibration. The deviation of data points from a straight line is a systematic effect yielding a bow shape that can qualitatively be explained by the action of creep accumulated over the extension steps. The striking observation that the bow is not observed for the contraction measurement points to a significant difference of the piezo response for contraction and extension.

To clarify if the observation is inherent to the piezo or an artefact of the sequence of measurements or of a lack of accommodation time between measurements, we repeated the measurement in reverse order and with an extended accommodation time of 190 min between extension and contraction. The corresponding results compiled in Figure 1c,d qualitatively yield a perfect reproduction of the previous measurement, while there is a difference in quantitative results slightly beyond the statistical error of individual measurements. Based on the last measurements that we consider to be the most reliable ones, the study yields a calibration factor of *S_f_* = −0.722 nm/V without specified error margins. We keep in mind that the response of the tube piezo depends in detail on the direction of the movement and that there are slight differences for the directional calibration factors 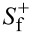 and 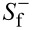. Principally, it might be possible to reduce the bow effect observed for the tube piezo extension by leaving more accommodation time between the voltage steps. However, in this case, thermal drift is likely to deteriorate the measurement by similar amounts as the observed creep.

The fact that the measured tube piezo calibration factor differs from the nominal value by more than a factor of two might be due to the manufacturing tolerance; but, more likely, it can be explained by a depolarization of the tube piezo material as it has been subject to many heating cycles for bakeout of the UHV chamber.

Next, we investigate the influence that the extension or extension history of the fiber tube piezo might have on the cantilever excitation. The calibration of the cantilever excitation system comprising the excitation piezo, the cantilever, their mutual coupling, and electrical system parameters, further termed amplitude calibration, is highly accurate when performed with a perfectly aligned interferometer. A high-precision measurement based on an expanded dataset as described in [13] yields a value of *S*^prec^ = (33.26 ± 0.27) nm/V for the amplitude calibration factor.

**Figure 2:**
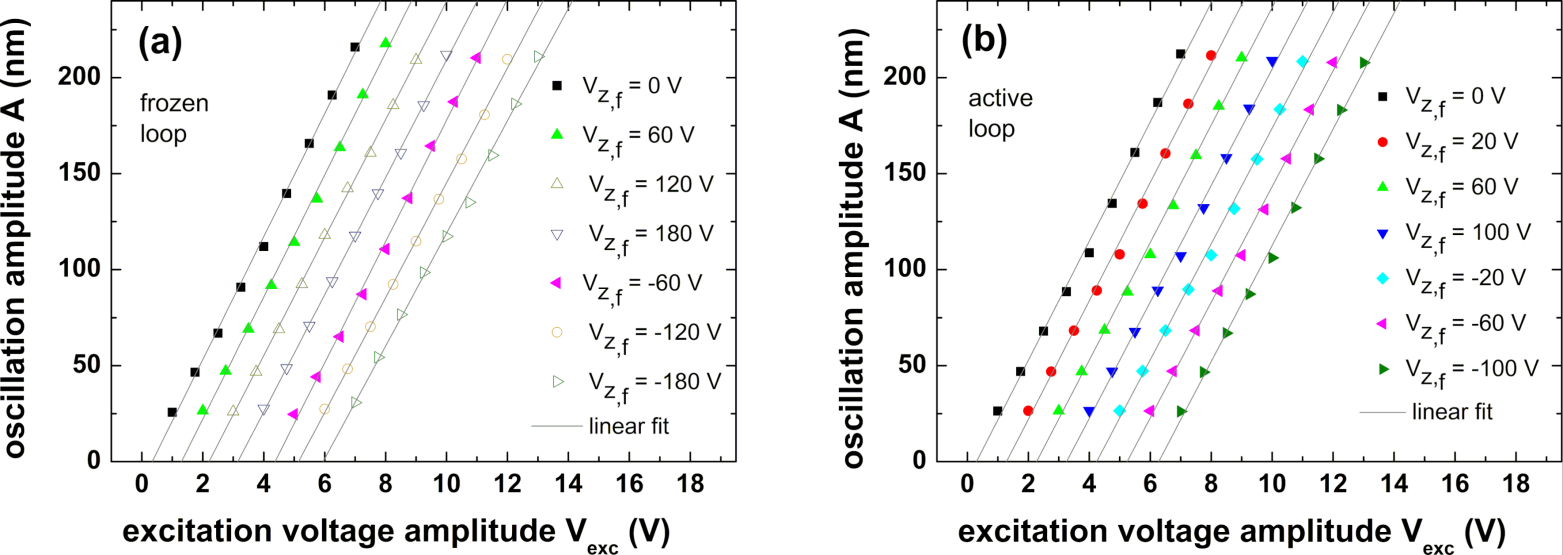
Cantilever excitation amplitude calibration performed for fiber tube piezo extension or contraction. Calibration measurements are started after adjusting the tube piezo *z* voltage *V_z,_*_f_ to the specified voltage and waiting for 10 min for system equilibration. (a) The interferometer is misaligned according to the applied negative and positive voltages. (b) The misalignment is compensated with a control loop. Note that data and fit lines are shifted by an increment of 1 V along the *V*_exc_ axis for each calibration to separate measurements from each other. The undulation of data points around the straight line is due to peculiarities in fitting the data as explained in [13].

For testing the calibration accuracy for the misaligned interferometer, various voltages 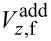 are applied to the fiber tube piezo, and, after each step of voltage change, the cantilever excitation voltage is varied from 1 V to 7 V in steps. For each step, the interferometer signal is analyzed to extract the oscillation amplitude *A* corresponding to the respective voltage step. Figure 2 shows plots of *A* against the cantilever excitation voltage *V*_exc_, where the data are fitted by straight lines. Note that all straight lines should coincide; however, they are shifted for each step along the *V*_exc_ axis for better clarity. The measurements presented in Figure 2a have been performed without activating the fiber piezo control loop. Hence, for each amplitude calibration run, the interferometer is misaligned by an amount defined by the respective voltage 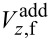. For the measurement represented in Figure 2b, the misalignment compensation loop has been activated. Consequently, the interferometer is forced back to the initial condition of perfect alignment before each run of amplitude calibration. To allow for piezo relaxation after significant extension or retraction, a waiting time of 10 min has been applied between the change in 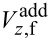 and the following calibration measurement.

The amplitude calibration factors determined with the aligned or misaligned interferometer are compiled in Table 1. The weighted mean of the calibration factors for the misaligned interferometer (frozen loop) is determined as 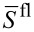 = (30.84 ± 0.15) nm/V, while in the measurements in which misalignment was corrected before each measurement (active loop), the mean of calibration factors is 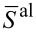 = (30.34 ± 0.24) nm/V.

**Table 1:** Measured calibration factors for the cantilever oscillation piezo determined for the non-aligned (frozen loop) and well-aligned (active loop) interferometer.

frozen loop		active loop	
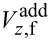	*S_i_* [nm/V]	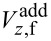	*S_i_* [nm/V]

0 V	32.008 ± 0.555	0 V	30.855 ± 0.679
60 V	31.794 ± 0.605	20 V	30.608 ± 0.662
120 V	30.736 ± 0.290	60 V	30.320 ± 0.661
180 V	30.542 ± 0.281	100 V	30.168 ± 0.634
−60 V	31.587 ± 0.537	−20 V	30.135 ± 0.638
−120 V	29.900 ± 0.387	−60 V	30.278 ± 0.672
−180 V	29.325 ± 0.560	−100 V	31.035 ± 0.501

For the misaligned interferometer (frozen loop), the maximum and minimum 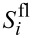 values for 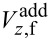 ≠ 0 differ by 2.470 nm/V and the 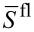 value for 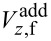 = 0 differs from the mean 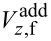 by 1.361 nm/V. Both differences are significantly larger than the standard deviation determined for the individual measurements and the weighted mean of uncertainties. This points to a systematic error in the amplitude calibration factor measurements. Applying the same analysis to the data of the alignment-corrected interferometer (active loop), we find that the respective differences are smaller or close to the relevant standard deviations. We conclude that, in the latter case, the loop action provides the same conditions for each calibration measurement, allowing for a calibration with 2% relative uncertainty in this case.

Above observations clearly demonstrate that the misalignment and the fiber piezo motion history may deteriorate the excitation system calibration. This is probably due to piezo hysteresis and the fit procedure that has been optimized for the perfectly aligned interferometer [13] but not extensively tested for the misaligned interferometer. The slight difference between the *S_i_* values corresponding to 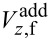 = 0 for frozen and active loops is a result of a slight change in the interferometer alignment during about 24 h elapsed between respective measurements. The differences between both values and *S*^prec^ are even larger as the precision measurement has been performed many weeks earlier. The difference in calibration results for measurements taken with significant time elapsed between can be explained by slight differences in the alignment of the laser spot on the cantilever due to manual adjustment or drift. Although, in our models, light is treated as a plane wave, in reality the light beam exiting the fiber end has a divergence of about 9°. As a consequence, light diffracted at the edges of the cantilever acts back on the reflected light sampled by the fiber. Furthermore, the partially coherent light of the laser produces speckle patterns [26] depending on minute charges in the beam profile or cantilever alignment. Both effects result in a considerable dependence of the lateral intensity distribution in the cantilever plane [25] on details of the alignment and, in turn, have an impact on the amount and interference structure of the reflected light, affecting the amplitude calibration.

## Conclusion

Our results demonstrate that a tube piezo, which is part of an interferometer for cantilever displacement detection, can precisely be calibrated by dynamic interferometry. However, care has to be taken in performing and analyzing experiments as piezo non-linearity and creep may have a considerable impact on the calibration results. Therefore, the calibration should be performed under conditions that are as close as possible to the conditions of the experiment the calibration results are used for. Generally, the system should be given much time to relax to avoid the deterioration of results by piezo creep. Also, one has to keep in mind that the piezo as a complex electromechanical system can be characterized by a single parameter *S*_f_ only in a limited range of system parameters. We find that, provided necessary precautions are applied, a valid amplitude calibration by dynamic interferometry can be performed for the well-aligned interferometer; however, additional care has to be taken for an amplitude calibration performed under conditions of a misaligned interferometer.

## Data Availability

Data generated and analyzed during this study is available from the corresponding author upon reasonable request.
